# Accelerating Crystal Structure Prediction with Machine Learning Forcefields

**DOI:** 10.1063/4.0001021

**Published:** 2025-10-27

**Authors:** Aaron D Kaplan

**Affiliations:** Lawrence Berkeley National Laboratory, Berkeley, CA, USA

## Abstract

Long-standing methods in materials simulation can now generally predict crystalline structure for near-/stable materials with high accuracy, and independently of local materials chemistry. However, these methods, particularly density functional theory, are electronic structure-based and scale unfavorably with material complexity. This has hindered study of, e.g., configurationally disordered and glassy materials. More, electronic structure calculations are typically run at zero temperature. To bridge the gap between non-universal atomistic simulation and electronic structure modelling, universal machine learning forcefields (MLFFs) have recently attained a level of accuracy suitable for rapid prediction of crystal structure with near electronic structure accuracy. In my talk, I'll discuss the materials databases such as the Materials Project [1] which are used to train these potentials. I will also discuss how the Materials Project's thermodynamic information, including competition between polymorphs, is crucial for determining ground-state crystal structure. I'll highlight recent advances in training universal MLFFs with purpose-built datasets like MatPES [2] which aim to make these models more efficient while retaining their level of predictive accuracy. I'll showcase a range of applications of MLFFs, including benchmarks for ground-state structure prediction, determining positions of hydrogen atoms, representing disordered crystals from ICSD, and extensions to non-crystalline / glassy-like materials through a simulated melt-quench process.
